# A novel closed technique for ultrasound-guided plantar fascia release with a needle: review of 107 cases with a minimum follow-up of 24 months

**DOI:** 10.1186/s13018-021-02302-y

**Published:** 2021-02-24

**Authors:** A. Iborra, M. Villanueva, P. Sanz-Ruiz, Antonio Martin, Concepción Noriega

**Affiliations:** 1 School of Health Sciences, Department of Podiatry, University of La Salle, Institute Avanfi, 28020 Madrid, Spain; 2 Institute Avanfi, 28020 Madrid, Spain; 3 Orthopaedic and Trauma Department, Hospital General Universitario Gregorio Marañón, Madrid, Spain; 4 Orthopaedic and Trauma Department, Hospital General Universitario Donostia, Madrid, Spain; 5 University of Alcalá, Madrid. School of Medicine and Health Sciences, Department of Nursery and Physiotherapy, University of Alcalá, Alcalá de Henares, Spain

**Keywords:** Plantar fasciitis, Heel pain, Needle, surgery, Ultrasound-guide surgery

## Abstract

**Abstract:**

**Background:**

This study aims to analyze the clinical outcome of a new ultrasound-guided surgery for partial plantar fasciotomy performed with a needle for treatment of plantar fasciitis.

**Methods:**

We performed a retrospective review of 107 patients diagnosed with plantar fasciitis who underwent ultrasound-guided release of the plantar fascia.

The series included 62 males (57.9%) and 45 females (42.1%) treated between April 2014 and February 2018, with a mean follow-up of 21.05 ± 10.96 months (7–66) and a minimum follow-up of 24 months. The mean age was 48.10 ± 10.27 years (27–72).

Clinical assessments and ultrasound examination were carried out before treatment, after 1 week, and then after 1, 3, 12, and 24 months. The clinical assessment was based on a visual analog scale and the Foot and Ankle Disability Index.

**Results:**

Heel pain improved in 92.5% (99) of patients, but not in 7.4% (8 patients). In the group of patients whose heel pain improved, 9 experienced overload on the lateral column and dorsum of the foot, which improved with the use of plantar orthoses and a rehabilitation program. We recorded no nerve complications (e.g., paresthesia), vascular injuries, or wound-related problems.

**Conclusion:**

Ultrasound-guided partial plantar fasciotomy with a needle is safe, since structures are under direct visualization of the surgeon and the risk of damage is minimal. Stitches are not necessary, and recovery is fast. Consequently, costs are low, and the patient can return to work quickly. This technique may represent a valid option for treatment of plantar fasciitis.

## Introduction

It is estimated that 10% of the population experience heel pain at some point in their lives, although few data are available from high-quality epidemiological studies [1]. Plantar fasciitis is the most common cause of lower heel pain in the United States, affecting more than 2 million people per year. Its high prevalence in the USA (approximately 4% to 7%) results in $284 million per year being spent on treatment [2]. The incidence of plantar fasciitis is greater among people aged between 40 and 60 years, with a female-to-male predominance of 2:1 [1, 3]. Plantar fasciitis accounts for 25% of all foot lesions in athletes, among whom it is also the most frequent cause of talalgia [4].

Plantar fasciitis is caused by degeneration of the plantar fascia resulting from repetitive microtears due to repeated traumatism or overload, rather than from a primary inflammatory reaction [5]. The cause of plantar fasciitis is currently unknown, although it is believed to be multifactorial, with abnormal biomechanics and delayed healing of collagen of the fascia as likely contributors [6].

Risk factors for plantar fasciitis include excessive foot pronation or flat feet, pes cavus, shortening of the Achilles tendon or gastrocnemius muscle, limb length discrepancy, obesity, overtraining, prolonged standing or walking, and improper gait [7, 8]. Most patients seek treatment within the first year, and in the vast majority of cases, pain resolves within the first year. Multiple treatments have been proposed in the medical literature, and most specialists choose to initiate conservative treatment, which is effective in 70–80% of cases. The most widely used approaches are physical therapy, plantar orthotics, gastrocnemius stretching, and corticosteroid injections [9, 10].

Surgery is considered 6 to 12 months after failure of nonsurgical management. One of the treatment options is partial plantar fascia release, which may be performed as open, percutaneous, and endoscopic or ultrasound-guided release. The technique involves resection of approximately 40–50% of the fascia to minimize the effect of instability on the arch and maintain the normal biomechanics of the foot.

Ultrasound-guided partial plantar fasciotomy was described by Vohra et al. in 2009 [11]. The authors performed the release with a tourniquet and a 0.5-cm incision to insert the instruments. They closed the incision with a 4-0 nylon suture, which is removed between 10 and 14 days after surgery. The technique described by the authors has since undergone modifications, the quality of the ultrasound equipment has improved, and surgery can be performed via Abbocath, with no need for a large incision.

Since 2014, we have modified our surgical approach and now perform ultrasound-guided plantar fasciotomy using multiple perforations with a needle. We then make a vertical windshield wiper movement with the bevel of the tip of the needle, which acts as a knife, thus enabling the release [12].

The aim of this study was to describe our new ultrasound-guided surgical technique and to carry out a retrospective review of outcomes, recovery times, and complications. Our preliminary clinical results show this approach to be as effective as other published techniques. Complications and recovery times are minimal, and costs are greatly reduced, since the procedure is performed in the office.

## Methods

Our retrospective study was performed in accordance with the principles of the 1964 Declaration of Helsinki (2013 revision) and approved by the Research Ethics Committee of our center (US-PH-COT-2014). All participants gave their informed consent to participate in the study and for their clinical and radiological data to be reproduced.

We carried out a retrospective review of 107 patients who underwent ultrasound-guided surgery for plantar fascia release between April 2014 and February 2018. The minimum postoperative follow-up was 24 months. The review included patients diagnosed with plantar fasciitis for whom conservative treatment had failed.

All the patients had experienced the classic symptoms of plantar fasciitis, including pain on taking the first few steps after waking or after prolonged sitting. Patients felt pain on palpation of the proximal plantar fascia and reported that the pain worsened as the day progressed.

**Fig. 1:**
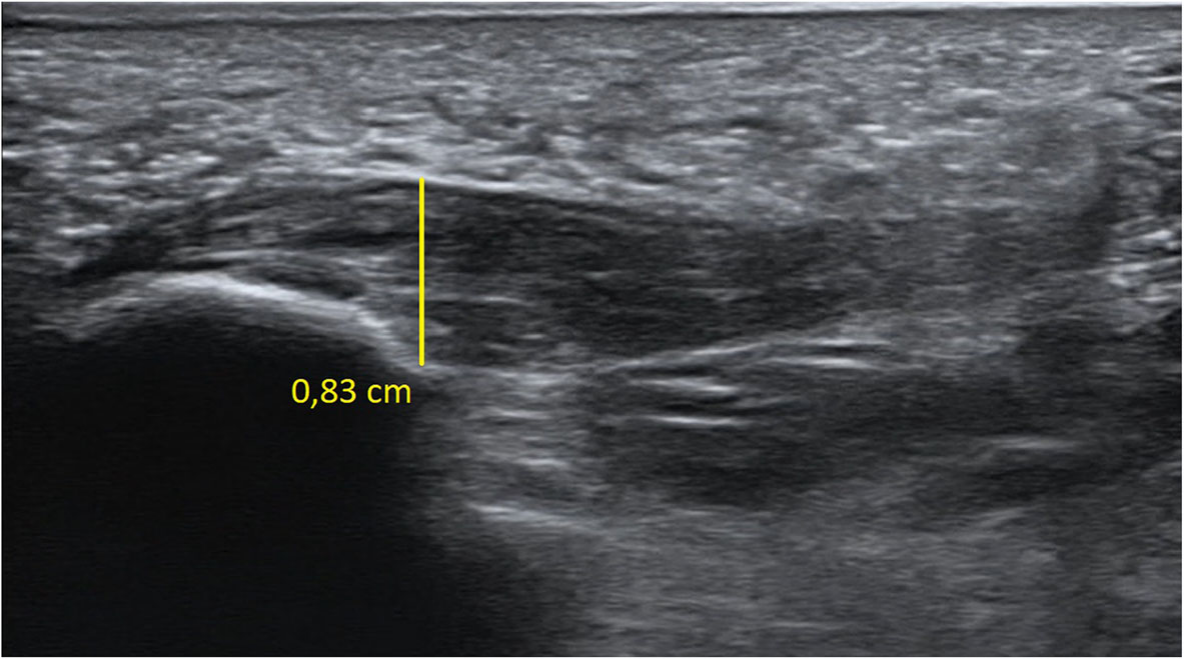
Hypoechoic and thickened plantar fascia: 0.83 cm

The diagnosis was established based on symptoms and confirmed by ultrasound using high-resolution equipment (Alpinion E15) with an 8- to 17-MHz linear multifrequency transducer. Plantar fasciitis was confirmed if the thickness of the fascia was greater than 0.4 cm, as described by McMillan et al, who reported a hypoechoic image in the area of the damaged fascia compared with the healthy fascia. Each measurement was repeated three times at each visit to avoid intra-observer bias [13] (Fig. 1).

To be included, patients had to be of legal age. Patients also had to have experienced plantar fasciitis in the proximal third of the plantar fascia for 6 months or more and have previously received conservative treatment. Patients with bilateral plantar fasciitis were excluded.

The exclusion criteria were talalgia that did not meet the diagnostic criteria for plantar fasciitis, bilateral plantar fasciitis, no conservative treatment for at least 6 months, not meeting the inclusion criteria, and plantar fasciitis associated with another condition such as nerve entrapment.

All patients underwent a clinical assessment and ultrasound assessment before treatment and after 1, 3, 12, and 24 months. The clinical assessment was based on a visual analog scale (VAS) (0–10) and the Foot and Ankle Disability Index (FADI), which assesses function on a scale of 0 to 100.

### Surgical technique

**Fig. 2:**
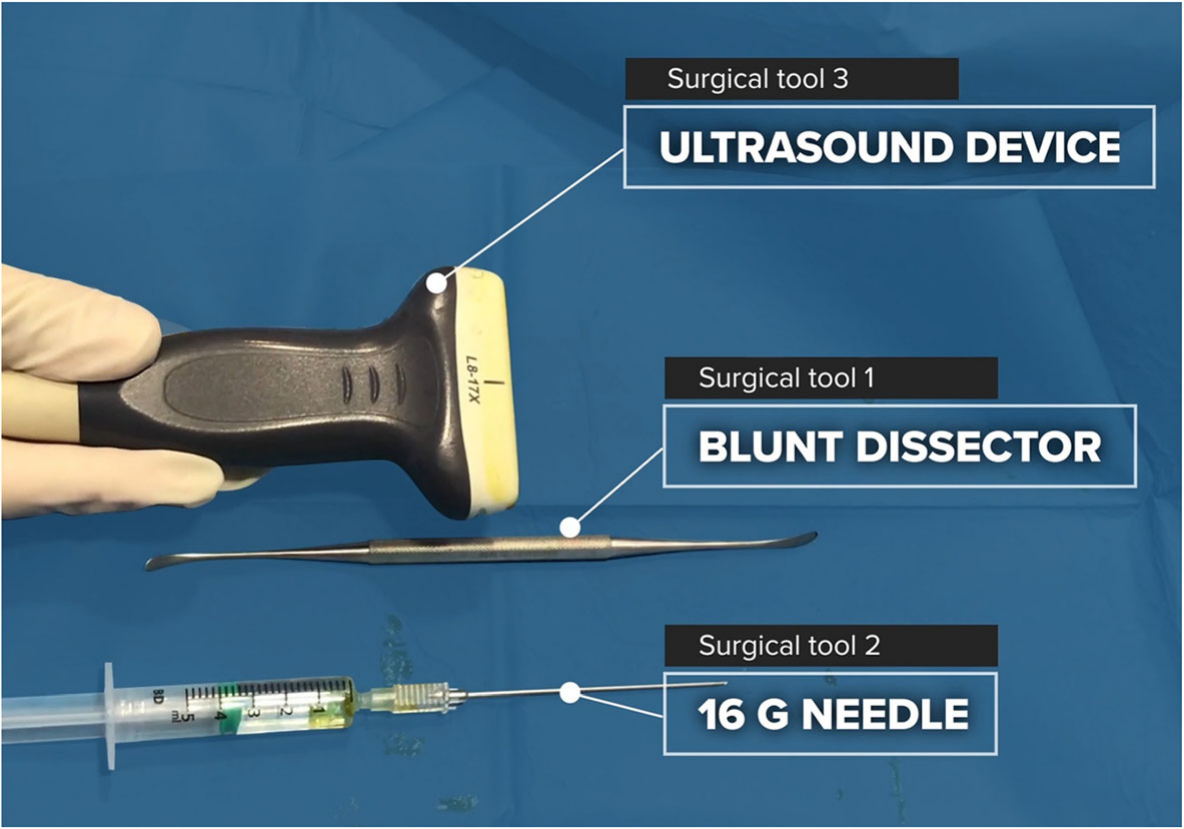
Instrument set

The instrument set of included a needle (16 G), a blunt dissector, an ultrasound device (Alpinion ECube15) with an 8- to 17-MHz linear transducer and the Needle Vision Plus™ software package (Alpinion Medical Systems, Bothell, WA, USA) (Fig. 2).

All patients underwent ultrasound-guided partial plantar fasciotomy using an Abbocath 16 G needle.

Patients were supine with the affected limb in external rotation and the contralateral leg in hip and knee flexion or in extension. The foot rested at the end of the examination table or hung over the edge.

**Fig. 3:**
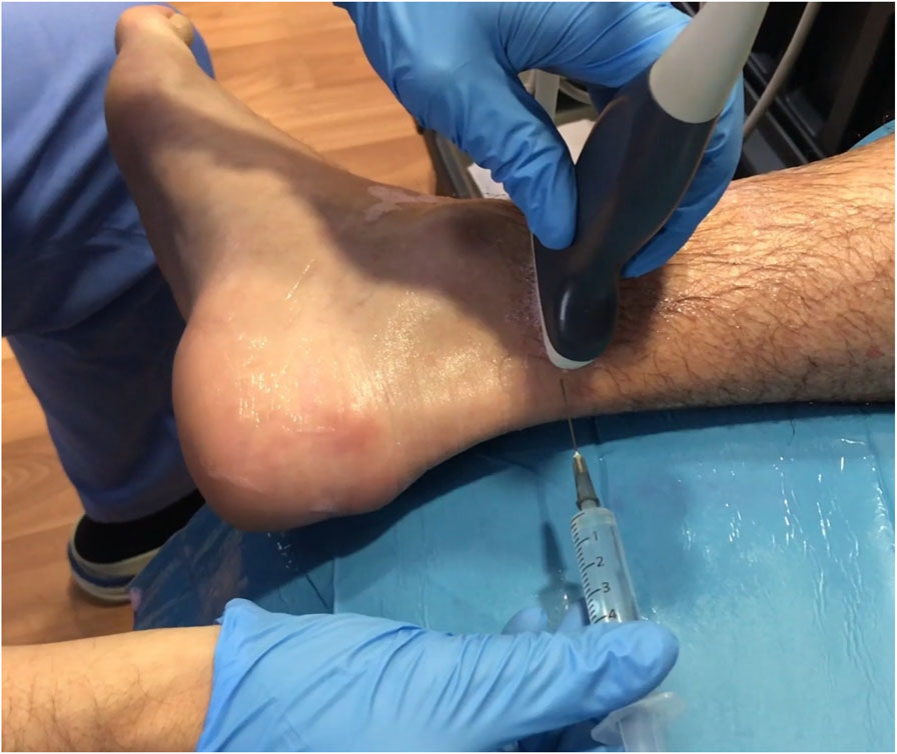
Ultrasound-guided tibial nerve block

The tibial nerve was identified in the ankle using ultrasound and then infiltrated under direct visualization with 2 to 3 ml of mepivacaine 2% (Fig. 3).

**Fig. 4:**
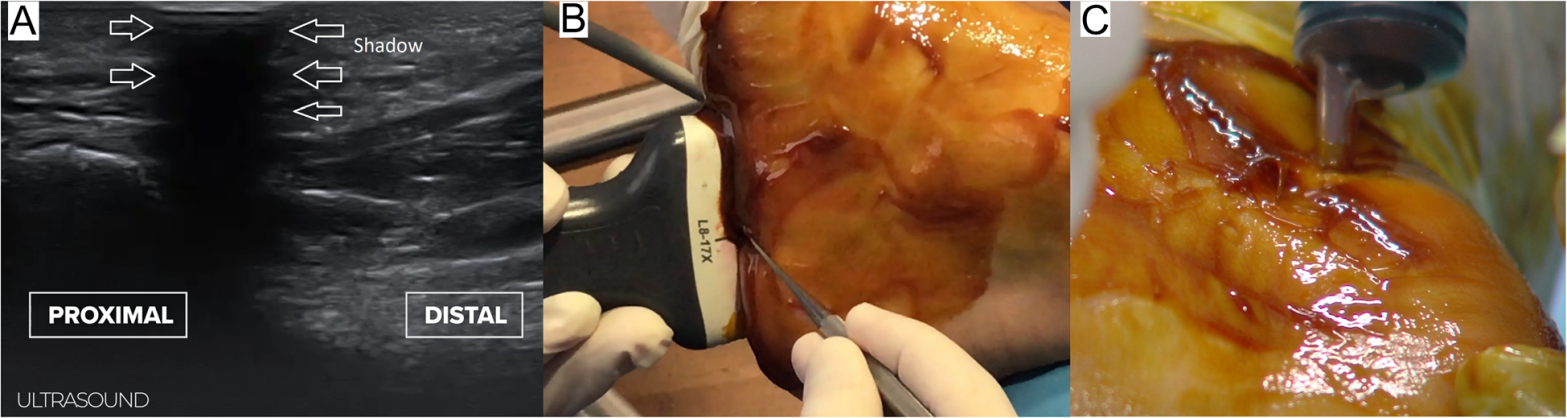
Acoustic shadows (**a**). Using a blunt dissector (**b**). Marking the skin with the hollow tip of a syringe (**c**)

With the ultrasound in both planes, we created acoustic shadows using a blunt dissector in order to determine the entry point. We marked the skin at the desired point by creating marks with the hollow circle of a syringe, thus avoiding damage to nerves or vessels (Fig. 4).

The surgical field was prepared with sterile material after disinfection of the whole foot with povidone-iodine or chlorhexidine.

**Fig. 5:**
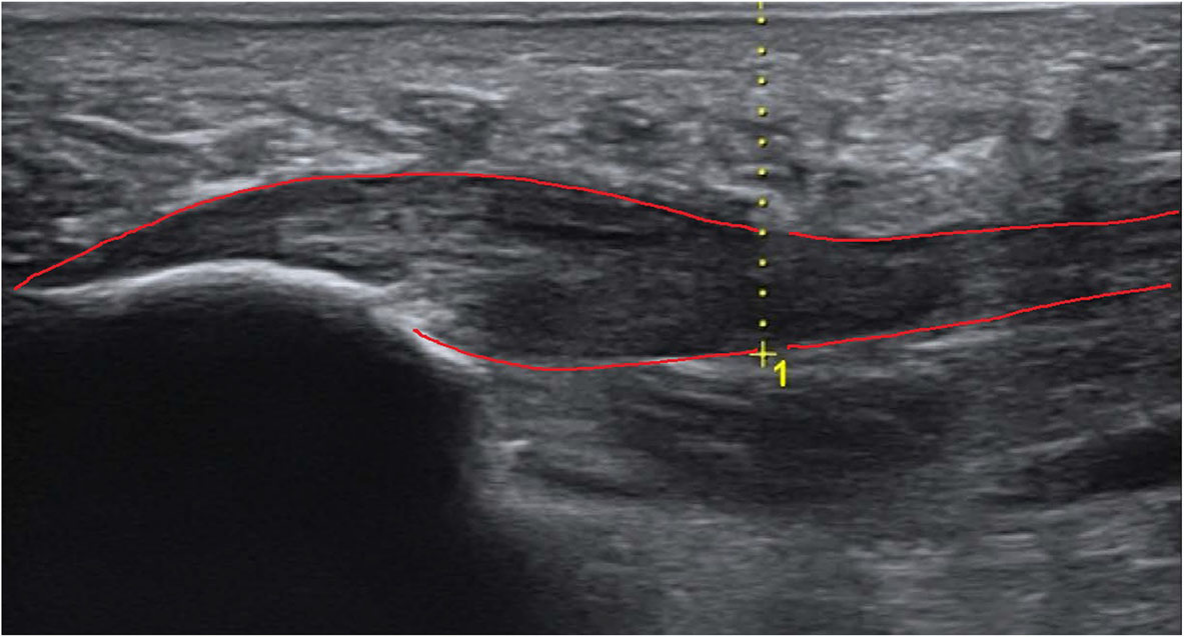
Selecting the entry point for partial plantar fasciotomy

With the probe in the longitudinal axis, we selected the entry point for the partial plantar fasciotomy, usually at the point where the thickness of the plantar fascia changes from thick to normal. The point selected may change from patient to patient (Fig. 5).

**Fig. 6:**
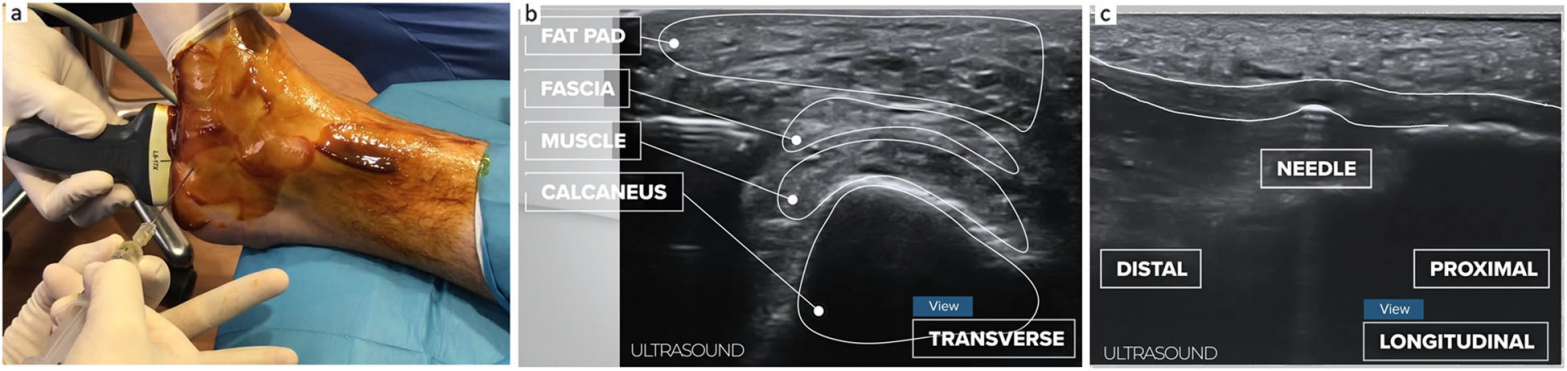
Insertion of the needle (**a**). Needle between fascia and muscle (**b**). Raising the plantar fascia with the needle (**c**)

The needle was under direct visualization at all times. At the chosen entry point, we inserted the 16 G needle from medial to lateral, with both the needle and the transducer transverse to the plantar fascia. The needle was then placed between the fascia and the muscle. At this point we checked our position, raised the plantar fascia, and verified that we were underneath it in both the longitudinal and the transverse views (Fig. 6).

With the ultrasound device in a transverse position, the needle was used to repeatedly perforate the plantar fascia from medial to lateral [12].

After the initial perforations, we started to move the needle in a windshield wiper motion, from deep to superficial and from medial to lateral, always in the same plane, in order to make a single linear cut. With this movement, the bevel of the Abbocath acts like a knife over the debilitated fascia, thus enabling controlled and fast advance of the cut, rather than weakening of the fascia, which can then tear with forced dorsal flexion of the foot and ankle. We tried to create the most linear cut possible by constant monitoring in both the transverse and the longitudinal planes.

**Fig. 7:**
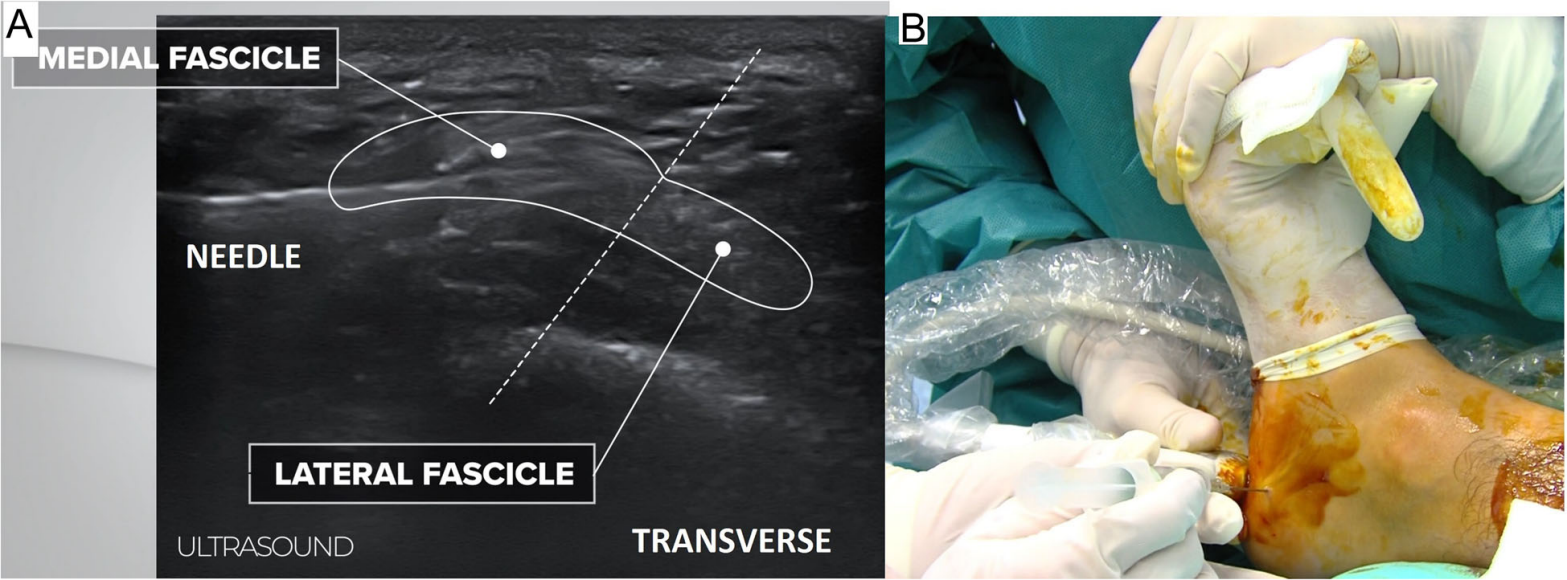
Perforation and windshield wiper motion to release the plantar fascia (**a**). Dorsal flexion of the toes and ankle (**b**)

During the perforations and the cutting movement, the assistant held the probe and maintained the foot and ankle in forced dorsal flexion in order to create tension in the fascia, thus making the release more effective (Fig. 7).

The release is checked by palpating with the needle, moving it from deep to superficial, and manually verifying the absence of tension. In addition, the process can be visualized directly using ultrasound in both the transversal and the longitudinal axes.

**Fig. 8:**
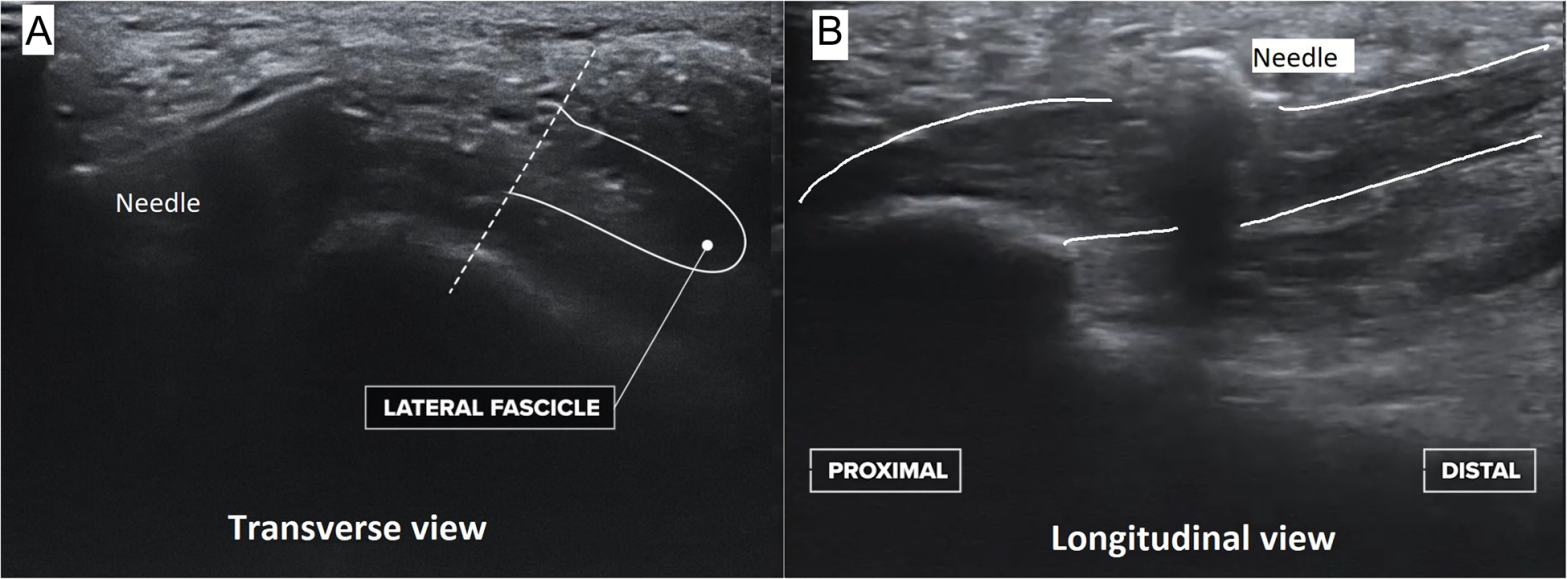
Transverse (a) and longitudinal (**b**) views of the procedure, showing how the needle disrupts the fibers throughout the thickness of the fascia

The needle passes easily from the most plantar area of the fascia, at its border with the muscle, to the plantar fat, thus showing that there are no fibers left to cut. We try to release no more than 50% of the width of the medial fascia (Fig. 8).

**Fig. 9:**
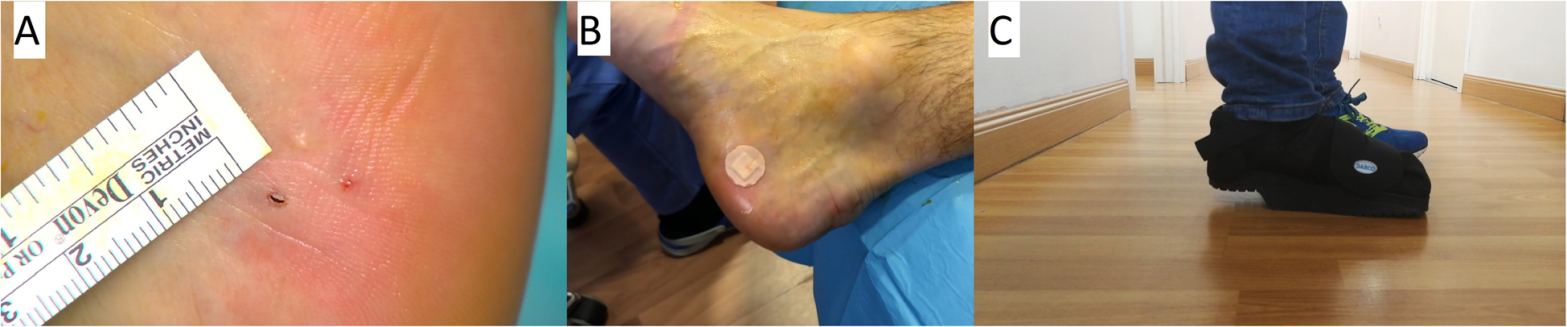
Incisions 1–2 mm (**a**), a small adhesive dressing (**b**), reverse heel post-surgery shoe (**c**)

After completion of the release, we inject 1 ml of bupivacaine 0.5% and 1 ml of betamethasone sodium phosphate (Celestone cronodose®) to reduce inflammation and pain. A small adhesive dressing is then placed over the needle entry point, thus ending the procedure. The procedure lasts 10–15 min. The patient leaves the clinic walking, with partial weight bearing and the help of a reverse heel post-surgery shoe. Low-molecular weight heparin and antibiotic therapy were not required (Fig. 9).

The statistical analysis was performed using R Ver. 5.3.1 (R Foundation for Statistical Computing, Institute for Statistics and Mathematics, Welthandelsplatz 1, 1020 Vienna, Austria). Statistical significance was set at *p* < 0.05. Qualitative variables are expressed as absolute values and frequencies; quantitative variables are expressed as mean and standard deviation. The Shapiro-Wilk test was used to determine the normality of the study variables, except for age, weight, height, and body mass index. The VAS and FADI results were analyzed using the Friedman test (omnibus) with post hoc tests and using the Wilcoxon signed-rank test with a Bonferroni correction. Effect size was defined using Kendall’s *W* as small (< 0.1), medium (0.10–0.25), and large (> 0.25). Thickness (cm) was analyzed using the Wilcoxon signed-rank test. The effect size was defined using r as small (< 0.4), medium (0.4–0.6), and large (> 0.6).

## Results

Mean age was 48.10 ± 10.27 years (27–72), and the average clinical course was 21.05 ± 10.96 months (7–66). There were 62 male patients (57.9%) and 45 female patients (42.1%). The median body mass index (BMI) was 25.34 ± 2.07 (range 19–32).

Foot posture was classed as neutral in 14 cases (13.1%), pronated in 72 (67.3%), highly pronated in 13 (12.1%), supinated in 5 (4.7%), and highly supinated in 3 (2.8%).

The results of the VAS (0–10) and the Foot and Ankle Disability Index (FADI) (0–100) in preoperative time and after 1 month, 3 months, and 12 months are shown in Table 1.

**Table 1:** VAS, FADI, fascia plantar thickness

	Preop	1 month	3 months	12 months	Difference (95% CI)
VAS	7.523 ± 0.817	1.692 ± 1.532	1.692 ± 1.532	0.944 ± 1.726	− 6.58 (− 6.91, − 6.21)
FADI	36.028 ± 12.786		86.405 ± 13.309	93.297 ± 13.641	57.3 (53.8, 60.3)
Thickness (cm)	0.655 ± 0.09			0.634 ± 0.081	− 0.0214 (− 0.033, − 0.0135)

**Fig. 10:**
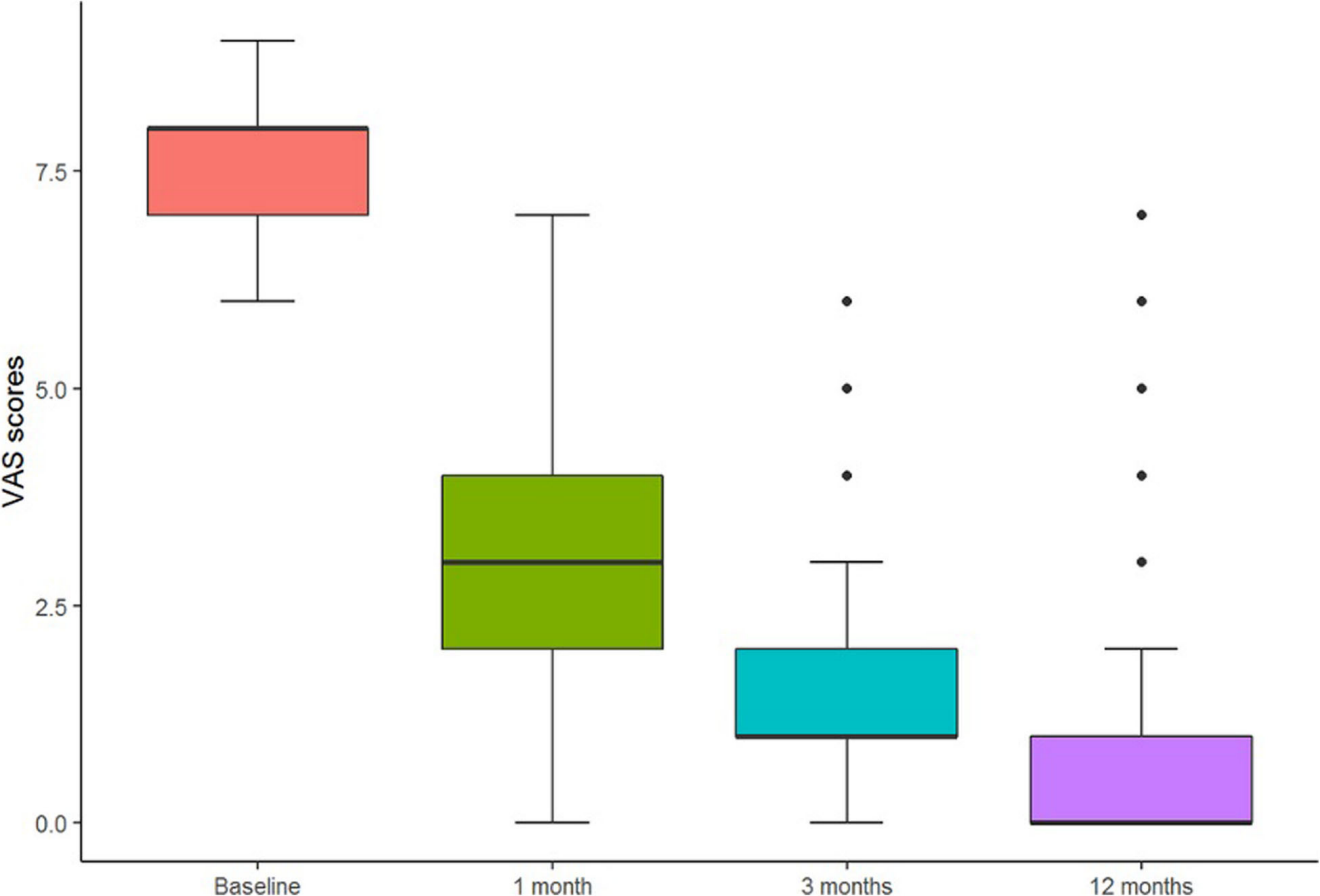
Visual analog scale (VAS)

**Fig. 11:**
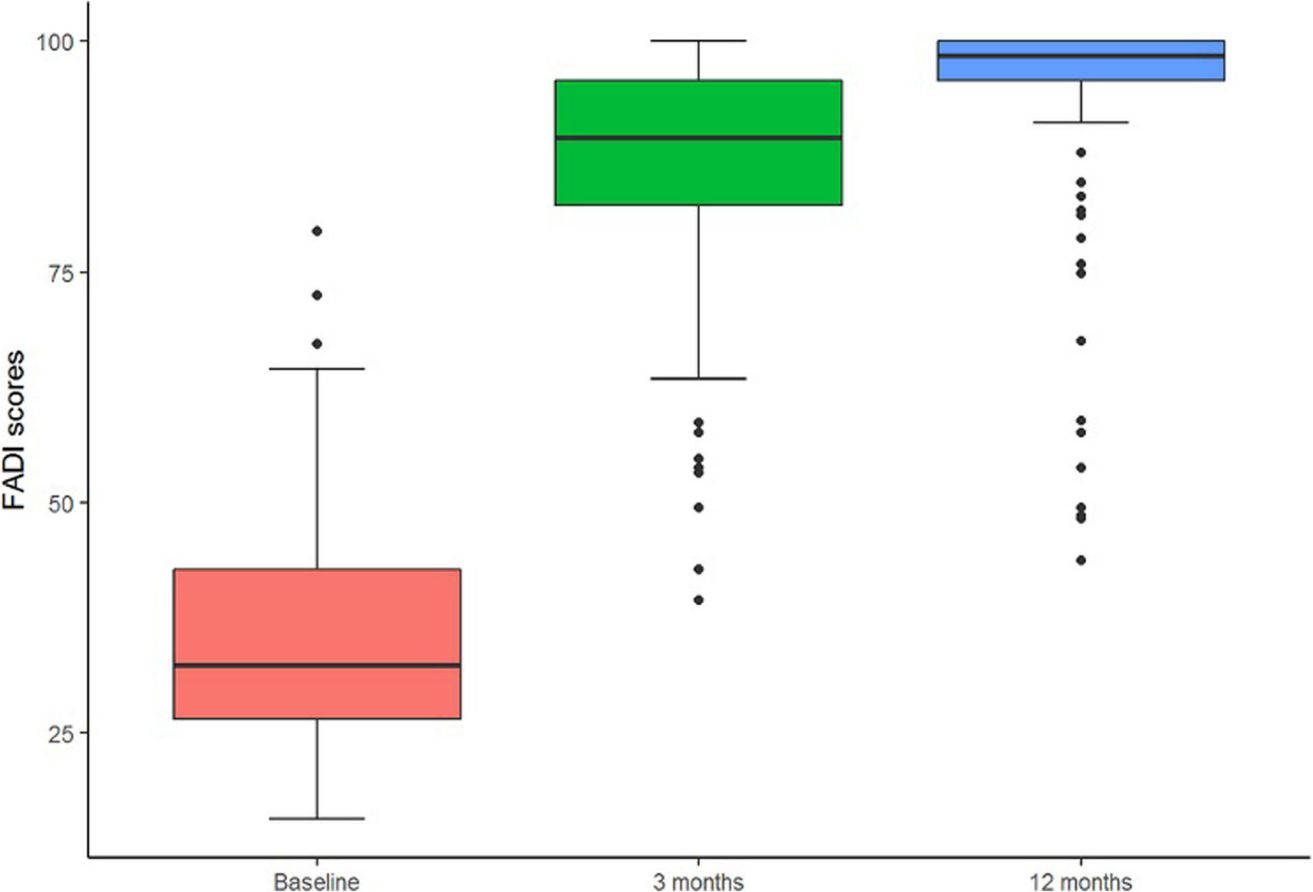
Foot and Ankle Disability Index (FADI)

The VAS score decreased significantly at all visits compared with baseline and compared with the previous visit (7.523 ± 0.817 to 0.944 ± 1.726), except between 3 and 12 months, where the decrease was not significant (Fig. 10, Table 1). The FADI questionnaire was administered before the technique and 3, 12, and 24 months after (Fig. 11, Table 1). Since there were no changes between 12 and 24 months, we did not include the 24-month assessment in the statistical analysis. The FADI increased significantly at all visits compared with baseline and the previous visit (6.028 ± 12.786% to 93.297 ± 13.641%).

The most pronounced decrease in the VAS score occurred during the first month after the procedure; the most pronounced decrease in the FADI occurred during the third month after the procedure (Table 1). Plantar fascia thickness decreased during the study from 0.655 ± 0.09 cm to 0.634 ± 0.081 cm, although without reaching normal values (Table 1), as reported elsewhere [13, 14].

There were significant differences in the **VAS score** (*χ* 2 [3] = 284.177, *p* < 0.001), with a large and significant effect size (Kendall’s *W* = 0.885 95%CI [0.829, 0.829]) and a decrease of − 6.58 (− 6.91, − 6.21) points. There were significant differences in the **FADI** (*χ* 2[2] = 188.585, *p* < 0.001), with a large and significant effect size (Kendall’s *W* = 0.881 95%CI [0.815, 0.815]) and an increase of 57.3 (53.8, 60.3) points.

Heel pain improved in 92.5% of patients (99); no improvement was observed in the remaining 7.4% (8).

## Discussion

Treatment of plantar fasciitis is generally successful with conservative measures in 70–80% of patients [15]. The high prevalence of this condition (approximately 4 to 7% of the population) means that $284 million is spent each year on treatments in the USA [2].

If conservative measures fail after 6 months, surgical release of part of the plantar fascia is indicated. The release can be performed as an open, percutaneous, endoscopic, or ultrasound-guided procedure [12, 16–18].

The results reported in the literature are considered good to excellent with open surgery in 84–92% of cases, with endoscopic fasciotomy in 82–92%, and with percutaneous surgery in 81–93% [16, 19–23].

The most commonly defined postoperative complications are loss of the medial longitudinal arch, forefoot pain, and medial and lateral column overload, in particular after complete medial plantar fasciotomy. Lateral column pain depends on the extent of the release of the plantar fascia. Fewer than 50% of patients are likely to develop this condition [24]. Potential postoperative complications include infection, injury to nerves or vessels, and reflex sympathetic dystrophy syndrome [25–27].

Percutaneous surgery is a minimally invasive technique, although to some extent it is a “blind procedure,” as the precise resection of the plantar fascia is not visible and there is a relative risk of damaging other structures [28].

Endoscopic partial fasciotomy has increased in popularity in recent years, as it improves recovery and complications of open surgery. This technique must be carried out with a tourniquet, since bleeding in tissues limits endoscopic vision. The main complication described with endoscopic surgery is injury to the posterior tibial nerve and its branches. Barrett et al. reported neurological complications, such as numbness of the fifth toe and damage to the lateral plantar nerve, although the frequency is very low [16, 29–31].

Ultrasound-guided surgery is a novel approach with proven indications, such as gastrocnemius lengthening, carpal tunnel release, tarsal tunnel release, and plantar fasciitis [12, 32–34]. Our technique is based on the concept of McShane et al., who described ultrasound-guided release of the carpal tunnel with an Abbocath [35], and can be considered a progression of the techniques previously described by the authors in 2014 with a scalpel and in 2016 with a needle. The main difference is that instead of multiple horizontal perforations, from medial to lateral, we use the bevel of the tip of the Abbocath as a knife, thus speeding up the procedure and enabling more accurate sectioning of the fibers.

The procedure shares all the advantages of previously reported approaches. In addition, it does not require a tourniquet and causes minimal skin damage. It can be performed in the office, much in the same way as an infiltration, thus avoiding the cost of an operating room.

The procedure requires a small incision (1 mm) with local anesthesia, thus reducing pain and speeding up recovery. We injected corticosteroids in combination with bupivacaine at the end of the procedure. This may be a confounding factor for VAS and function, as it may help to reduce pain and swelling. However, we do not consider it affected the final outcome, as all patients were injected with corticosteroids before the surgical procedure, without response. In our series, the largest decrease in the VAS was recorded during the first month after the procedure, and the largest decrease in the FADI was recorded during the third month (Table 1).

As ultrasound enables the fascia to be viewed at all times, a precise release of no more 40–50% of the width can be performed. The risk of biomechanical alterations due to excessive release of the plantar fascia is minimized, and damage to other structures is avoided [36].

Our results are similar to those reported in the literature with open surgery, endoscopic fasciotomy, and percutaneous surgery. We observed an improvement in heel pain in 92.5% of patients [99] and no improvement in 7.4% [10]. Twelve patients experienced external column overload, probably due to alteration of the biomechanics of the foot.

Patients whose heel pain improved also experienced an improvement in lateral column pain with the use of a custom plantar orthosis; however, plantar orthosis did not resolve lateral column pain in patients whose condition did not improve.

Eight out of 107 patients in our series did not improve after partial fasciotomy with a needle. Five reported that their pain was the same as before surgery and three patients reported the same pain plus an overload of the lateral column that was not resolved.

The thickness of the plantar fascia was analyzed before and after surgery and decreased throughout the follow-up. However, the difference was not significant, and in no case did the plantar fascia regain its normal thickness. Therefore, we can conclude that pain resolves even though the thickness of the plantar fascia does not return to normal.

We did not observe complications affecting the nerves, such as paresthesia at the entry portal, or vascular lesions [16, 30, 31]. Some patients had small hematomas, which resolved in 2 weeks.

In our experience, ultrasound-guided partial plantar fasciotomy using multiple perforations is a safe technique with very satisfactory results that reduces recovery time and work absenteeism. The technique can be performed on patients with underlying conditions such as diabetes, vascular insufficiency, and heart disease.

## Conclusion

Ultrasound-guided plantar fasciotomy with a needle is an easy technique with whose preliminary results are similar to those reported for other techniques. Although prospective randomized studies are necessary, the theoretical advantages of this procedure include a reduction in nerve complications, elimination of hospital costs, and potentially faster recovery.

### Note

A video illustrating this surgical technique has been included in the Educational Media Program of the American Academy of Orthopaedic Surgeons (AAOS) and is available for surgeons upon request

## Data Availability

The materials described in the manuscript, including all relevant raw data, are available from the first author upon request by e-mail.

## Abbreviations

Mhz: Megahertz
FADI: Foot and Ankle Disability Index
VAS: Visual analog scale

## References

[1] McNally EG, Shetty S (2010). Plantar fascia: imaging diagnosis and guided treatment. Semin Musculoskelet Radiol..

[2] Tong KB, Furia J (2010). Economic burden of plantar fasciitis treatment in the United States. Am J Orthop Belle Mead NJ..

[3] Rasenberg N, Bierma-Zeinstra SM, Bindels PJ, van der Lei J, van Middelkoop M (2019). Incidence, prevalence, and management of plantar heel pain: a retrospective cohort study in Dutch primary care. Br J Gen Pract J R Coll Gen Pract..

[4] Taunton JE, Ryan MB, Clement DB, McKenzie DC, Lloyd-Smith DR, Zumbo BD (2002). A retrospective case-control analysis of 2002 running injuries. Br J Sports Med..

[5] Lemont H, Ammirati KM, Usen N (2003). Plantar fasciitis: a degenerative process (fasciosis) without inflammation. J Am Podiatr Med Assoc..

[6] Lee WCC, Wong WY, Kung E, Leung AKL (2012). Effectiveness of adjustable dorsiflexion night splint in combination with accommodative foot orthosis on plantar fasciitis. J Rehabil Res Dev..

[7] Luffy L, Grosel J, Thomas R, So E (2018). Plantar fasciitis: a review of treatments. JAAPA Off J Am Acad Physician Assist..

[8] Petraglia F, Ramazzina I, Costantino C (2017). Plantar fasciitis in athletes: diagnostic and treatment strategies. A systematic review. Muscles Ligaments Tendons J..

[9] Moshrif A, Elwan M (2019). The effect of addition of buffered dextrose 5% solution on pain occurring during local steroid injection for treatment of plantar fasciitis: a randomized controlled trial. Muscle Ligaments Tendons J..

[10] Allam AE, Chang K-V (2020). Plantar Heel Pain. StatPearls.

[11] Vohra PK, Japour CJ. Ultrasound-guided plantar fascia release technique: a retrospective study of 46 feet. J Am Podiatr Med Assoc. 2009;99(3):183–90. 10.7547/0980183 19448167

[12] Iborra A, Villanueva MJ, Barret SL. Ultrasound-guided plantar fascia release with needle: a novel surgical technique. Open J Orthop. 2016;6(7):159–70.

[13] McMillan AM, Landorf KB, Barrett JT, Menz HB, Bird AR (2009). Diagnostic imaging for chronic plantar heel pain: a systematic review and meta-analysis. J Foot Ankle Res..

[14] Liang H-W, Wang T-G, Chen W-S, Hou S-M (2007). Thinner plantar fascia predicts decreased pain after extracorporeal shock wave therapy. Clin Orthop..

[15] Chen C-M, Chen J-S, Tsai W-C, Hsu H-C, Chen K-H, Lin C-H (2013). Effectiveness of device-assisted ultrasound-guided steroid injection for treating plantar fasciitis. Am J Phys Med Rehabil..

[16] Barrett SL, Day SV, Pignetti TT, Robinson LB (1995). Endoscopic plantar fasciotomy: a multi-surgeon prospective analysis of 652 cases. J Foot Ankle Surg Off Publ Am Coll Foot Ankle Surg..

[17] Çatal B, Keskinbora M, Keskinöz EN, Tümentemur G, Azboy İ, Demiralp B (2019). Percutaneous Plantar Fascia Release With Needle: Anatomic Evaluation with Cadaveric Specimens. J Foot Ankle Surg Off Publ Am Coll Foot Ankle Surg..

[18] Cheung JT-M, An K-N, Zhang M (2006). Consequences of partial and total plantar fascia release: a finite element study. Foot Ankle Int..

[19] Apóstol-González S, Herrera J (2009). Percutaneous surgery for plantar fasciitis due to a calcaneal spur. Acta Ortop Mex..

[20] Davies MS, Weiss GA, Saxby TS (1999). Plantar fasciitis: how successful is surgical intervention?. Foot Ankle Int..

[21] Fallat LM, Cox JT, Chahal R, Morrison P, Kish J (2013). A retrospective comparison of percutaneous plantar fasciotomy and open plantar fasciotomy with heel spur resection. J Foot Ankle Surg Off Publ Am Coll Foot Ankle Surg..

[22] Stone PA, McClure LP (1999). Retrospective review of endoscopic plantar fasciotomy. 1994 through 1997. J Am Podiatr Med Assoc..

[23] Tountas AA, Fornasier VL (1996). Operative treatment of subcalcaneal pain. Clin Orthop..

[24] Brugh AM, Fallat LM, Savoy-Moore RT (2002). Lateral column symptomatology following plantar fascial release: a prospective study. J Foot Ankle Surg Off Publ Am Coll Foot Ankle Surg..

[25] Boyle RA, Slater GL (2003). Endoscopic plantar fascia release: a case series. Foot Ankle Int..

[26] Hogan KA, Webb D, Shereff M (2004). Endoscopic plantar fascia release. Foot Ankle Int..

[27] Lundeen RO, Aziz S, Burks JB, Rose JM (2000). Endoscopic plantar fasciotomy: a retrospective analysis of results in 53 patients. J Foot Ankle Surg Off Publ Am Coll Foot Ankle Surg..

[28] Oliva F, Piccirilli E, Tarantino U, Maffulli N (2019). Percutaneous release of the plantar fascia. New surgical procedure. Muscle Ligaments Tendons J.

[29] Thomas ZM, Thomas KJ (2017). Technique Tip: Single-Incision Endoscopic Plantar Fasciotomy. Foot Ankle Spec..

[30] Komatsu F, Takao M, Innami K, Miyamoto W, Matsushita T (2011). Endoscopic surgery for plantar fasciitis: application of a deep-fascial approach. Arthrosc J Arthrosc Relat Surg Off Publ Arthrosc Assoc N Am Int Arthrosc Assoc..

[31] Ogilvie-Harris DJ, Lobo J (2000). Endoscopic plantar fascia release. Arthrosc J Arthrosc Relat Surg Off Publ Arthrosc Assoc N Am Int Arthrosc Assoc..

[32] Iborra A, Villanueva M, Sanz-Ruiz P (2020). Results of ultrasound-guided release of tarsal tunnel syndrome: a review of 81 cases with a minimum follow-up of 18 months. J Orthop Surg..

[33] Villanueva M, Iborra Á, Rodríguez G, Sanz-Ruiz P (2016). Ultrasound-guided gastrocnemius recession: a new ultra-minimally invasive surgical technique. BMC Musculoskelet Disord.

[34] Villanueva M, Iborra Á, Ruiz MDM, Sanz-Ruiz P (2019). Proximal ultrasound-guided gastrocnemius recession: a new ultra-minimally invasive surgical technique. J Foot Ankle Surg Off Publ Am Coll Foot Ankle Surg..

[35] McShane JM, Slaff S, Gold JE, Nazarian LN (2012). Sonographically guided percutaneous needle release of the carpal tunnel for treatment of carpal tunnel syndrome: preliminary report. J Ultrasound Med Off J Am Inst Ultrasound Med..

[36] Tweed JL, Barnes MR, Allen MJ, Campbell JA (2009). Biomechanical consequences of total plantar fasciotomy: a review of the literature. J Am Podiatr Med Assoc..

